# An Insight into Sesamolin: Physicochemical Properties, Pharmacological Activities, and Future Research Prospects

**DOI:** 10.3390/molecules26195849

**Published:** 2021-09-27

**Authors:** Reny Rosalina, Natthida Weerapreeyakul

**Affiliations:** 1 Graduate School (Biomedical Sciences Program), Faculty of Pharmaceutical Sciences, Khon Kaen University, Khon Kaen 40002, Thailand; renyrosalina@kkumail.com; 2 Division of Pharmaceutical Chemistry, Faculty of Pharmaceutical Sciences, Khon Kaen University, Khon Kaen 40002, Thailand; 3 Human High Performance and Health Promotion Research Institute, Khon Kaen University, Khon Kaen 40002, Thailand

**Keywords:** sesamolin, sesame lignan, *Sesamum indicum* L., pharmacological activity, physicochemical properties, physicochemical enhancement

## Abstract

Sesame seeds are rich in lignan content and have been well-known for their health benefits. Unlike the other sesame lignan compounds (i.e., sesamin and sesamol), the study of the pharmacological activity of sesamolin has not been explored widely. This review, therefore, summarizes the information related to sesamolin’s pharmacological activities, and the mechanism of action. Moreover, the influence of its physicochemical properties on pharmacological activity is also discussed. Sesamolin possessed neuroprotective activity against hypoxia-induced reactive oxygen species (ROS) and oxidative stress in neuron cells by reducing the ROS and inhibiting apoptosis. In skin cancer, sesamolin exhibited antimelanogenesis by affecting the expression of the melanogenic enzymes. The anticancer activity of sesamolin based on antiproliferation and inhibition of migration was demonstrated in human colon cancer cells. In addition, treatment with sesamolin could stimulate immune cells to enhance the cytolytic activity to kill Burkitt’s lymphoma cells. However, the toxicity and safety of sesamolin have not been reported. And there is also less information on the experimental study in vivo. The limited aqueous solubility of sesamolin becomes the main problem, which affects its pharmacological activity in the in vitro experiment and clinical efficacy. Therefore, solubility enhancement is needed for further investigation and determination of its pharmacological activity profiles. Since there are fewer reports studying this issue, it could become a future prospective research opportunity.

## 1. Introduction

Sesamolin is the commonly known furofuran lignan isolated from the seeds of *Sesamum indicum* L. [1,2]. Sesame was first cultivated 4000 years ago, and is thus considered as one of the most ancient crops to produce oil [3]. The total annual production of sesame in the world is about 5,532,000 metric tons (MT), with 50% from Asia and 30% from Africa [4]. Sesame seeds contain 50% oil, 25% protein, and the rest are sugars, moisture, fibers, and minerals, and the majority of the sesame lignans including sesamolin, sesamin, sesamol, and sesaminol are found in sesame seeds and the oils [5,6].

The health benefits of sesame seeds were largely contributed by its lignans content such as sesamin, sesamol, and sesamolin. Several recent reviews have presented the pharmacological activity of sesame oils in in vitro and in vivo experiments; some of them also prefer to focus on the pharmacological effect of sesame lignans compound like sesamol or sesamin [7,8,9,10]. Sesamolin, one of the major sesame lignan compounds, has been reported to possess antioxidant, neuroprotective, and anticancer activities. Notwithstanding, the report related to exploration in pharmacological activities of sesamolin is limited.

Along with the activities, several reports reveal the physicochemical limitations of sesamolin that can be the major drawbacks of their pharmacological activities. Sesamolin has limited water-solubility that causes it to be categorized as class II in Biopharmaceutic Classification System, which is a class for low water solubility and high permeability compounds. The compound belonging to this class needs physicochemical properties improvement, especially the solubility profile, to improve its pharmacological effect and to be developed as a drug candidate [11,12]. This issue may become the main obstacle to research sesamolin’s pharmacological activities, yet this may become a research opportunity to enhance the physicochemical properties of sesamolin in order to improve the therapeutic effect. Therefore, this review presents the summary of the information on the recent update research on sesamolin in terms of major source, identification, and purification method, the physicochemical properties, and pharmacological activities of sesamolin with its mechanism of action. Moreover, the limitation related to the physicochemical properties of sesamolin and the future research prospects in the associated field were also reviewed.

## 2. Source and Sesamolin Content in Sesame

Sesame (*Sesamum indicum* L.), of the Pedaliaceae family, is the major source of sesamolin and other lignan compounds including sesamin, sesamol, sesaminol, sesamolinol, and glycosylated-lignans. Although other sesame lignans such as sesamin were reported to be isolated from other plant species like *Piper* sp., *Virola* sp., *Magnolia* sp., and *Camellia* sp., recent updates showed that no reports of sesamolin have been isolated from other plant families than Sesamum. However, other species of Sesamum such as *S. angustifolium*, *S. alatum*, *S. radiatum*, *S. angolense* Welw., *S. calcynum* Welw., and *S. orientale* var. *malabaricum* Nar. were reported to also contain sesamolin in small quantity [1,7,13].

Several studies have reported that the sesamolin content in sesame seeds generally ranged from 0.2–4.3 mg/g dried seeds as shown in Table 1. In the majority, sesamolin content was lower than sesamin, while sesamol was the least constituent among the three lignans. However, lignan content proportions in different sesame cultivars might vary. Several factors such as varieties, seeds color, geography, and growth conditions of cultivation could affect the phytoconstituents in sesame seeds. Korean black sesame cultivars had higher sesamolin contents than sesamin, yet the average lignan content of Korean white sesame was higher. This study also found that the lignan content was significantly different between two crop years (2009 and 2010), indicating that environmental stress and agronomic conditions influenced the lignan content [14]. In contrast to those findings, Indian black sesame cultivars contain the highest total lignan content, and white sesame cultivars contain high sesamol content. A high total lignan content in black sesame seeds, also reported by Shi et al. in sesame seeds cultivated in China [15,16]. A study in the landrace and breeding line of sesame from Thailand showed a wide range of sesamolin content, between 0–2.25 mg/g. Landrace sesame seeds, Maehongsong, had a higher level of sesamolin than sesamin. However, the A7250-8 and A7251-7 (BR) breeding lines did not contain any sesamolin [17].

Sesamolin content in sesame oils could be affected by the oil processing process. Oil’s processing technologies generally have two different processes. The first is when the seed is roasted, and the second is when the crude oil is refined. Thus, there are some various sesame oils products based on those oils processing, (1) hot-pressed sesame oil (HPSO), and small mill sesame oil (SMSO) use roasted seeds, (2) cold-pressed sesame oil (CPSO) uses non-roasted seeds, and (3) refined sesame oil (RSO) uses either roasted or non-roasted seeds following by refining process. Roasted sesame seed oils (HPSO and SMSO) have a lower sesamolin level than that of the CPSO (unroasted seeds). The roasting process of sesame seeds may cause the oxidation of sesamolin converted into sesamol, resulting in low sesamolin content. Meanwhile, sesamolin can be fractured into sesamol during the bleaching process. Thus, low sesamolin was also observed in RSO [15,18].

**Table 1:** Content of sesamolin in sesame seeds.

Country	Seeds Variation	Extraction Solvent	Quantification Method	Sesamolin (mg/g)	Sesamin (mg/g)	Sesamol (mg/g)	References
-	Commercial sesame seed	Hexane/isopropanol (3:1, *v*/*v*)	HPLC-Fluorescent	0.21–2.97	0.7–7.12	-	[5]
-	Commercial sesame seed	Methanol	HPTLC	0.59–1.48	1.13–2.83	-	[19]
Korea	White seed (2009)	90% Acetonitrile	HPLC-DAD-ESI/MS	2.95–3.72	3.24–5.53	-	[14]
	White seed (2010)			2.70–4.29	2.57–5.20	-	
	Black seed (2009)			1.12–2.29	0.87–1.97	-	
	Black seed (2010)			1.21–2.26	0.84–1.98	-	
China	Yellow seed	Methanol	HPLC-UV/VIS	0.20–2.32	1.11–3.29	n.d.–0.12	[15]
	Black seed			1.06–3.35	1.98–9.41	n.d.–0.07	
	Brown seed			1.0–2.46	1.54–4.75	n.d.	
	White seed			1.18–2.29	1.26–3.83	n.d.–0.15	
India	White seed	80% Ethanol	HPLC-UV/VIS	1.69–3.52	2.25–5.86	0.35–3.24	[16]
	Brown seed			1.52–3.43	2.10–5.98	0.16–0.63	
	Black seed			2.91–3.76	3.33–4.97	0.30–0.74	
Thailand	Landrace and breeding line sesame	80% Methanol	HPLC-DAD	n.d.–2.25	n.d.–7.23	-	[17]

n.d. = not detected.

## 3. Sesamolin Separation, Determination, and Purification Method

Sesamolin and other compounds in sesame can be identified qualitatively and quantitatively using various separation techniques followed by spectroscopy techniques for analysis. Before analyzing the compounds in sesame seeds or oil samples, preliminary preparation to eliminate interfering compounds and concentrate the lignans is needed. Various extraction methods such as solid-phase extraction and liquid-liquid extraction have been well-known methods for this purpose. Solid-phase extraction using solid sorbents graphene oxide and hydroxylated ferrous ferric oxide (Fe_3_O_4_) was successfully applied for sesame oil preparation prior to sesamolin, sesamin, and sesamol determination using high-performance liquid chromatography (HPLC) giving 85−93% recovery [20]. Ultrasonic-assisted liquid-liquid micro-extraction using deep eutectic solvent (DES) composed of choline chloride and *p*-cresol with the help of sonication for sesame oil extraction gives high extraction efficiency for polar and nonpolar lignans [21].

Among separation and identification using chromatography techniques, HPLC using ultraviolet (UV/VIS) detector, photodiode array (PDA) detector, or fluorescent detector is the most widely used method for separation and quantification of compounds due to its high sensitivity [7,15,17,22,23]. Besides, thin layer chromatography (TLC), gas chromatography (GC) coupled with a mass spectrometer (MS) provides good separation and reliable determination. Alternatively, the use of high-performance thin-layer chromatography (HPTLC) offers the rapid and cost-effective determination of lignan compounds in sesame compared to the HPLC, which is considered as a time-consuming method. Recently, the HPTLC method using a less harmful solvent successfully showed comparable results with the HPLC-DAD [19,24]. Recently, the Near-infrared spectroscopy (NIRS) analytical technique coupled with the chemometric analysis has provided a non-destructive, rapid, and eco-friendly compound determination. NIRS successfully predicted the concentrations of sesamolin and sesamin in sesame seeds close to the results from HPLC techniques [25,26].

Sesamolin can be purified from sesame seeds or oil extracts by various chromatography methods such as silica gel column, counter-current chromatography, preparative HPLC, and centrifugal partition chromatography. The other methods are crystallization and resin absorption. The silica gel column, followed by semi-preparative HPLC, successfully separated sesamolin and sesamin from sesame oils with high purity (>97%), but was low in yield [23,27]. Reshma and co-workers used crystallization to isolate the sesame oils lignan achieving a high quantity (54% yield) and 94.4% purity of sesamolin [28].

Separation and purification of sesamolin and sesamin from sesame seeds using the Countercurrent chromatography (CCC) method by employing petroleum ether (60−90 °C), ethyl acetate, methanol, and water 1:0.4:1:0.5 (*v*/*v*) as solvents system successfully obtained sesamolin with 64% recovery and 98% purity [29]. Hamman also found the separation of sesamolin and sesamin from sesame oil qualitatively when using CCC following with GC/MS method to separate many vegetable oils minor lipids components [30].

Most problems in compound isolation from plant oils sample were the removal of the triacylglycerol, which were >90% in oils before the separation process to enrich the targeted compounds. To achieve this goal Gournet and co-workers used resin absorption XAD-4 as a preliminary step to obtain a mixture almost free from sugars and polar lipids, then used the Fast Centrifugal Partition Chromatography (FCPC) to separate lignan components in sesame seeds extracts [2]. By using Centrifugal Partition Chromatography (CPC), sesamolin with 93% purity was successfully isolated from sesame seeds extracts and this method can be used with a high quantity of sample, which has never been reported previously [31]. In the recent report, Michailidish et al. also successfully separated the sesamin and sesamolin in sesame oils with high yield and high purity using centrifugal partition extraction (CPE), followed by centrifugal partition chromatography (CPC) using biphasic solvents system n-hexane/ethyl acetate/ethanol/water in proportion of 2:3:3:2 (*v*/*v*/*v*/*v*) [32].

## 4. Physicochemical Properties of Sesamolin

**Figure 1:**
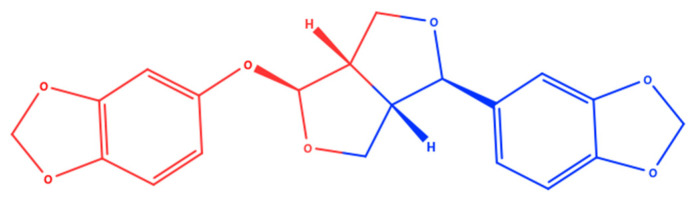
Sesamolin molecular structure.

Sesamolin has the molecular formula C_20_H_18_O_7_, and its chemical structure is shown in Figure 1. Sesamolin is in a group of lignan compounds formed from the uniting of two phenylpropanoids connected by the central carbon of their propyl side. The presence of methylene dioxyphenoxy moieties or its metabolite form—the phenolic hydroxyl group—might be responsible for the various biological activities of sesamolin [8]. However, no study reported the structure-activity relationship of sesamolin regarding which functional group is the pharmacophores for its biological activity.

The physicochemical properties of sesamolin are summarized in Table 2. The important physicochemical properties that affect the pharmacokinetic and pharmacodynamic behavior of the compounds are solubility, lipophilicity, hydrogen bond donors (HBDs), hydrogen bond acceptors (HBAs), and topological polar surface area (TPSA), Sesamolin has water-solubility less than 0.1 mg/mL that is considered practically insoluble in water. Aqueous solubility is an important property for bioactive compounds because it can affect activity in the in vitro and the in vivo assays, even in the clinical stages. At the in vitro experiment level, most of the in vitro tests used an aqueous medium, especially when using the cell model. The test compound must be completely dissolved in the medium at the adjusted concentration to evaluate its pharmacological effect. Furthermore, in the in vivo assay, the compound must be maintained at a specific concentration under the aqueous condition in order to be well-distributed via the bloodstream and provide high bioavailability to give the pharmacological effect at the target site [33].

The existence of hydrogen bond donors (HBDs) and hydrogen bond acceptors (HBAs) in compound structures contributes to its aqueous solubility, membrane absorption, and ligand-receptor interactions [34]. Sesamolin possesses less than 5 HBD and 2 to 16 HBA which is the optimum number for membrane absorption and provides sufficient interaction via hydrogen bond based on the Lipinski rule of five. The degree of lipophilicity of the compound is expressed as the coefficient partition (log P) and its important properties that define the absorption via the phospholipid bilayer. Sesamolin has log P value 3. A degree of lipophilicity value less than 5 is necessary for the compound to possess satisfactory absorption into membrane cells. The polar surface area (PSA) of the bioactive compound is required to bind with most of the target receptors. The polar surface area (PSA) of the bioactive compound determines its absorption. High PSA will increase solubility in water, but a PSA value of more than 140 Å will reduce the drug’s ability to permeate cells. The PSA of sesamolin is 64.6 Å, so it is considered to have good permeability [35,36,37].

## 5. Pharmacological Activities

### 5.1. Antioxidant Activity

Sesame seed is well known to have high antioxidant activity. Instead of the individual effect of the lignan compounds, the synergistic effect of tocopherol and lignans content in sesame contribute to the antioxidant activity of sesame [8]. Sesamolin showed low antioxidant activity in the various in vitro experiments. Sesamolin was found to exert lesser antioxidant activity than sesamol based on scavenging ability against DPPH radical and superoxide-free radical [38,39], ferrous reducing ability power (FRAP), oxygen radical absorbance capacity (ORAC), β-carotene-bleaching assay, and the inhibition of linoleic acid peroxidation [40]. However, the latter two antioxidant effects were higher than sesamin [40].

**Figure 2:**
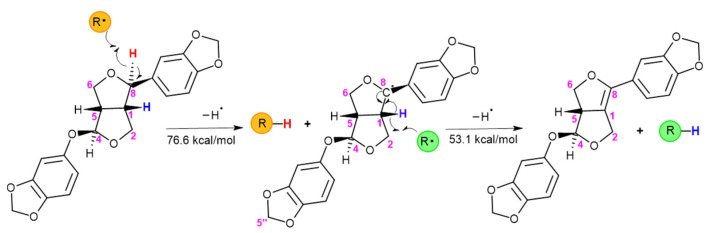
The proposed mechanism of sesamolin stabilized the free radical via hydrogen atom transfer based on Bond Dissociation Energy value. The abstraction of allylic hydrogen atom at C-8 to free radical generates sesamolin radical and stabilizes radical compound. Then the donation of a hydrogen atom at C-1 of sesamolin radical forms a double bond leading to the stabilization of the derived compound. Thus, sesamolin was able to donate two hydrogen atoms. The picture was drawn using MarvinSketch version 21.1.

The low antioxidant activity of sesamolin in vitro could be mainly due to the lack of the phenolic hydroxyl group, a good electron provider to free radicals. The possible mechanism of antioxidant activity of sesamolin was proposed via hydrogen atom transfer from the allylic hydrogen atoms at C-8 based on density functional theory (DFT) by computational study and C-H bond dissociation enthalpy (BDE) values (Figure 2). Therefore, sesamolin was predicted to possess a weaker antioxidant capacity than sesamin, which can donate two allylic hydrogens, and sesamol, which has a phenolic hydroxyl group [41]. Despite having weak antioxidant activity in the in vitro system, several studies have reported the antioxidant activity of sesamolin in vivo. Sesamolin did not inhibit lipid peroxidation activity of rat liver microsomes induced by ADP-Fe^2^^+^/NADPH in vitro. Sesamolin was found to inhibit lipid peroxidation of the rat liver and kidney after feeding with an extract containing 1% sesamolin. This activity was proposed to be from the metabolic conversion of sesamolin into two active metabolites, sesamolinol and sesamol [42]. The antioxidant activity of sesamolin in vivo was supported by the other study. Sesamolin possessed an inhibitory effect through the only microsomal system in the system using rat liver microsomes and cumene hydroperoxide (CumOOH)/Fe^2^^+^-ADP-NADPH, but not in a non-enzymatic system containing rat liver mitochondria and Fe^2^^+^-ascorbate [43]. This study also revealed the synergistic effect of individual lignans including sesamolin, sesamin, and sesamol with α-tocopherol or tocotrienol generated a higher inhibitory effect in both lipid peroxidation systems [43].

### 5.2. Antimicrobial Activity

Sesamolin has antimicrobial activity against *Bacillus cereus*, *Staphylococcus aureus*, and *Pseudomonas aeruginosa* with 61, 62, and 53 % growth inhibition at 2 mg/mL [40].

### 5.3. Neuroprotective Activity

Pathophysiology of neurodegenerative diseases was primarily associated with the biochemical alteration of biomolecules components in neuronal cells induced by oxidative stress. It is indicated by the excessive generation of reactive oxygen species (ROS) such as hydrogen peroxide, superoxide, and hydroxyl free radicals due to imbalance conditions between ROS and antioxidants leading to biomolecules damage [44]. The fact is, the brain, which is an important organ of the central nervous system (CNS) is highly vulnerable to oxidative stress [45]. The reduction of ROS can be a potential target for neurodegenerative disease prevention and treatment. Since ROS can be scavenged and attenuated by antioxidants, compounds that possess antioxidant activity can be the potential agents for the prevention and treatment of neurodegenerative disease therapy.

Several studies have evaluated the effect of sesamolin on protective activity in neuronal cells. Sesamolin successfully protected murine BV-2 microglial cells from hypoxia-induced cell death and hydrogen peroxide-induced cell injury [46,47]. Hypoxia for 1 h induced 35% cell death in the untreated group. Sesamolin 50 µM successfully increased cell viability to 96%, followed by decreasing LDH release by 24%. Moreover, sesamolin scavenged 25% of hypoxia-induced ROS in cells. Hypoxia-induced ROS may activate signal transduction pathways for cell death, including extracellular signal-regulated protein kinases (ERK1/2), c-Jun NH_2_-terminal kinase (JNK), and p38 Mitogen-activated protein kinases (MAPK). This study confirmed that the MAPK cascades were inhibited by sesamolin via preventing the phosphorylation of JNK, p38 MAPKs, and caspase-3 expression in BV-2 cells at 10 min hypoxia. Using different cells, the study in the protective effect of sesamolin was also reported by Hou in rat pheochromocytoma (PC12), and rat primary cortical cells [48]. They found that sesamolin reduced LDH release under hypoxia, which was correlated with the inhibition of MAPKs and caspase-3. Furthermore, hypoxia-induced apoptotic-like cell death, as detected by a fluorescent DNA-binding dye in cultured cortical cells, was reduced significantly after treatment with 50 µM sesamolin.

As well as ROS, activation of microglial cells will release nitric oxide (NO), of which overproduction can be toxic to neurons. Transcription of inducible-NO synthase (iNOS) genes in microglia regulated the NO generation in microglial by stimulation of lipopolysaccharide (LPS) that activates a complex array of intracellular signaling pathways involving tyrosine kinases, MAPK and NF-kB mediated gene expression. This stimulation induced the release of tumor necrosis factor (TNF-α) and facilitated neuron death. In vitro studies that utilized sesamolin to inhibit NO-induced by LPS confirmed that sesamolin significantly reduces the excess generation of LPS-induced NO in the murine microglial cell line BV-2 and rat primary microglial cells via the reduction of LPS-induced p38 MAPK [49].

The neuroprotective effects of sesamolin were performed in vivo using gerbils. Prior to focal cerebral ischemia induction, the gerbils were orally administered with purified sesamin or a crude sesame oil extract containing 90% sesamin and 10% sesamolin 20 mg/kg/day for 4 days. Sesamin and sesame extract containing sesamolin significantly reduced infarct sizes of gerbil brains in cerebral ischemia by 56% and 49%, respectively (*p* < 0.05). However, the mechanism of in vivo neuroprotection was not fully understood [50].

Neurodegenerative disease, especially Alzheimer’s disease (AD) indicated accumulation of proteins including extracellular amyloid plaques (Aβ) and neurofibrillary tangles (NFT) in the brain. Sesamolin’s protective effect against the toxicity of Aβ was evaluated using worm (*Caenorhabditis elegans*) models, which expressed the human Aβ fragment in the body wall muscle and was characterized by a progressive paralysis. In addition, the deposit of Aβ in neurons leads to attenuation of chemotaxis behavior. Sesamolin at a concentration of 100 µg/mL exhibit a significant delay of paralysis by 1.83 h in the transgenic worms. This value was higher than that of *Ginkgo* *biloba* leaf extract. Moreover, investigation on protective effect of sesamolin against Aβ toxicity in the neuronal cells using *C. elegans* CL2355 that expressed neuronal Aβ showed that the chemotaxis behavior was improved compared to the untreated group [51].

### 5.4. Antimelanogenesis

Melanogenesis is a process of melanin production occurring naturally in human skin as photoprotection from UV exposure but also causes pigmentation in the skin, as melanin is a dark brown color. Consequently, it will reduce the aesthetical value of the skin. Melanogenesis involves an interaction between keratinocytes and melanocytes. The process begins when keratinocytes are exposed to UV from sunlight and further activate the pro-opiomelanin genes, leading to the generation of α-melanocyte-stimulating hormone (α-MSH). α-MSH then binds with melanocortin-1 receptor (MC1R) on melanocytes. This engagement activates the signaling pathway via cyclic adenosine monophosphate (CAMP) and triggers the activation of Protein Kinase-A (PKA). The signaling continues with the upregulation of cAMP response element-binding (CREB) protein transcription factors, then promotes microphthalmia-associated transcription factor (MITF), resulting in the upregulation of transcription protein tyrosinase, TRP-1, and TRP-2, which are involved in melanin synthesis. Biochemical synthesis of melanin occurs in melanosomes beginning from the hydroxylation of tyrosine to 3,4-dihydroxyphenylalanine (L-DOPA), followed by oxidation to *o*-dopaquinone, then dopachrome is catalyzed by tyrosinase. Finally, eumelanin formation (dark brown color) occurs via enzymatic transformation of dopachrome by TRP-1 and TRP-2 [52,53].

The UV protective effect and anti-melanogenesis activity and the sunscreen function of sesamolin were evaluated in comparison to the well-established depigmenting agents, kojic acid, and β-arbutin. This study confirmed that sesamolin possessed sunscreen function by primarily absorbing UVB and exhibited 4-fold higher absorbance than kojic acid and β-arbutin. Although sesamolin showed low inhibition in mushroom tyrosinase, a key enzyme in melanogenesis, it showed high inhibition up to 50% in cellular tyrosinase at concentration 50 µg/mL compared with kojic acid and β-arbutin without causing any toxicity in noncancerous Vero and melanoma SK-MEL2 cell lines. Sesamolin at 25 µg/mL decreased the melanin content in SK-MEL2 cells. Western blot assay showed that sesamolin was downregulating the expression of tyrosinase, TRP-1, and TRP-2 in the SK-MEL2 cell line. This study suggests that sesamolin could inhibit melanin synthesis via two stages; (1) protected from UV radiation, the melanin inducer, via sunscreen function, and (2) downregulated the melanogenic protein tyrosinase, TRP-1, and TRP-2 [54].

**Figure 3:**
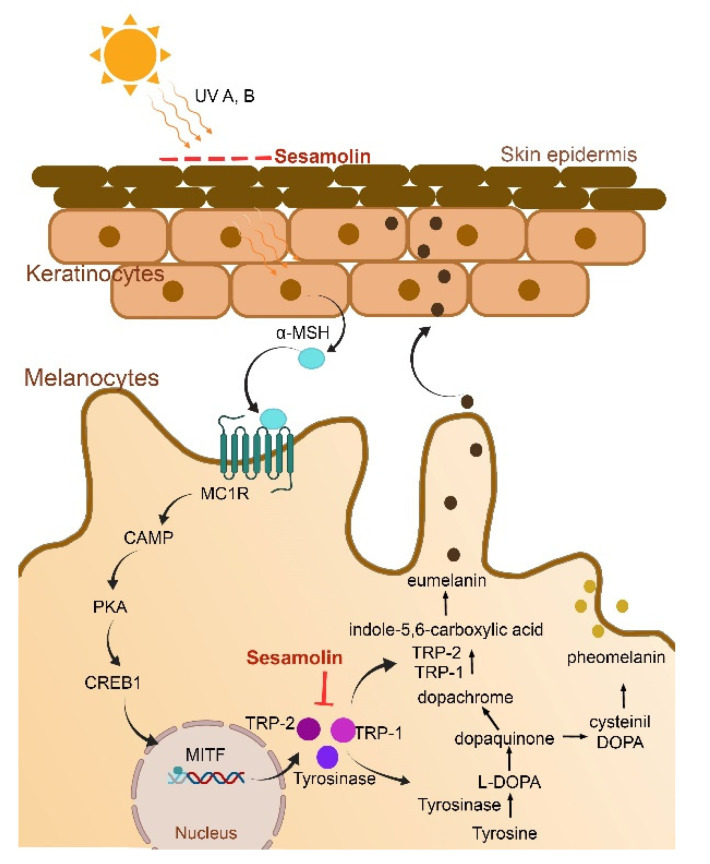
Sesamolin was able to inhibit melanin production via two mechanisms. (1) by blocking the UV radiation, which is the melanin inducer as sunscreen function (2) by downregulate the key melanogenic protein, tyrosinase, TRP-1 and TRP-2. The figure was created using Adobe Photoshop version 22.3.0.

The anti-tyrosinase activity of sesamolin was also reported by Michaildish based on the inhibition of mushroom tyrosinase activity in vitro. The results showed that sesamolin exerted moderate anti-tyrosinase activity at 500 μΜ and weak activity at 100 and 25 μΜ [32]. Sesamolin also showed high antimelanogenesis activity in skin cancer cells (B16F10). This study evidenced that sesamolin inhibited the expression of melanogenesis-related mRNA levels, as well as proteins such as tyrosinase and TRP-1 and TRP-2 at a concentration of 50 μΜ [55]. Figure 3 shows the summary of the mechanism of sesamolin inhibition of melanin production.

### 5.5. Anticancer Activity

Sesamolin showed growth inhibition and apoptosis induction in human lymphoid leukemia (Molt 4B) cells. Antiproliferation was a concentration-dependent manner with an IC_90_ of 90 µM. Sesamolin-induced apoptosis indicated by the morphological changes, DNA fragmentation, and formation of apoptotic bodies after 3 days of treatment with 90 µM sesamolin. When compared with other compounds in the sesame oil, episesamin, and sesamol from other studies, the growth inhibition of sesamolin was more effective. However, this study did not present a detailed mechanism of the apoptosis induction pathway or DNA fragmentation [56].

Effects of sesamolin on proliferative inhibition activity were also evaluated against human colon cancer HCT116. Antiproliferation based on MTT assay showed that sesamolin significantly inhibits the proliferation in a time-dependent manner and significantly inhibits migratory ability. Proliferation, differentiation, and apoptosis of cancer cells were regulated by the Janus kinase 2 (JAK2) signal transduction and activator transcription-3 (STAT3) signaling pathway. Sesamolin 20 µM significantly reduced the expression of p-JAK2/STAT3 indicated by the reduction of the p-JAK2/STAT3 band on western blot. Sesamolin and AG490 (a positive control) showed a synergistic effect. Their combination significantly downregulated the expression of p-STAT3. Cancer cell migration is a provision for metastasis, and it correlates with the upregulation of MMP 1, 2, and 9. This study showed that sesamolin downregulated MMP expressions in HCT116 when investigated by qRT-PCR. Sesamolin is a potential antiproliferative agent for colon cancer by inhibiting the activation of the JAK2/STAT3 pathway and prevent the cell invasion via inhibition of IL-6-induced expression of MMPs [57].

Another study investigated sesamolin for its anticancer activity in blood cancer Burkitt’s lymphoma cells, Raji by improving NK cell lysis activity [58,59]. The NK cell is one of the immune cells which has the ability to identify and distinguish normal and cancer cells then killing tumor cells. The killing activity (cytolysis) is triggered by the activation of activating receptors in NK cells, primarily NKG2D, by NKG2D ligands (NKG2DLs). The ULBP-1, ULBP-2, ULBP-3, MIC-A, and MIC-B were the NKG2DLs whose expression was gradually up-regulated by the progression of cancer on the cell surface. Conversely, normal cells have low expression of NKG2DLs. Therefore, the NKG2D receptors in NK cells can use NKG2DLs to easily recognize cancer cells in the surrounding normal tissue. The binding of activated NKG2D receptor in NK cells with NKG2DLs expressed in cancer cells resulting in a signaling pathway to release cytokine and induce cytotoxicity to kill the tumor cells. However, the NKG2DL levels decreased in late-stage tumors, and thus, decrease the sensitivity of cancer cells towards NK cells resulting in low cytolysis activity. Moreover, some cancer cells were reported to have naturally low expression of NKG2DLs, such as Ramos, Hep3B, and Raji [60,61]. For this reason, enhancing one or both expression of NKG2D in immune cells and NKG2DLs in tumor cells might modulate the antitumor immune response and could be a promising targeted therapy against cancers.

Utilization of sesamolin and sesamin to escalate NKG2DLs expression to improve NK cells-mediated cytolytic activity was reported by Kim in the human Burkitt’s lymphoma cell line (Raji), which has low sensitivity towards NK cells [58]. Pretreatment of Raji cells with 40 µM sesamolin for 72 h successfully elevated the sensitivity towards NK cells, resulting in the increase in cytotoxicity compared to the untreated group. Moreover, it was confirmed that the increase in cytolysis was followed by escalation of NKG2DLs expression ULBP-1, ULBP-2, and MICA/B in Raji cells. The increase of the ERK phosphorylation band in western blot assay and the weakened cytotoxicity on ERK inhibitor blocking assay proved that stimulation of the ERK signaling pathway by the sesamolin was involved in the escalation of NKG2DLs expression.

Besides targeting NKG2DLs, the NK cells-mediated cytolytic activity enhancement can be achieved by upregulating NKG2D receptor expression in NK cells. To investigate the direct effect of sesamolin on NK cells, both NK cells (NK-92MI) and Raji cells were treated with sesamolin. The cytolytic activity was increased in sesamolin-treated NK-92MI cells and on sesamolin-treated Raji cells compared to the untreated group. Consequently, when treated both Raji and NK-92MI cells with sesamolin, the increasing cytolytic activity of NK cells was also observed. The highest cytotoxicity of sesamolin against Raji and NK-92MI cells was at 20 μg/mL, and 40 μg/mL, respectively. The escalated expression of a membrane marker in the degranulation of the NK cells during cytolytic activity (CD107a) was observed in sesamolin-treated NK-92MI cells in a concentration and time incubation-dependent manner. Moreover, this study confirmed that expression of NKG2D in NK cells was elevated after NK-92MI was treated with 40 μg/mL for 72 h. Sesamolin triggered the phosphorylation of the p38, ERK1/2, and JNK pathways in NK cells to enhance the cytolytic activity [59]. The effect of sesamolin on cytolytic activity by governing the immunological responses against cancer cells was further investigated in dendritic cells (DC) [62]. The study indicated that sesamolin stimulated DCs to boost the killing and migratory activities of NK cells in the co-culturing of DCs and NK cells. The pharmacological activities of sesamolin and its mechanism of action are summarized in Table 3.

## 6. Pharmacokinetics

Further investigation of the pharmacological activity in the in vivo model using individual sesamolin has not been widely explored. Several studies have used animal models to study the pharmacological activity of sesamolin and other lignans in sesame seeds or oils. However, they did not report the pharmacokinetic profile of sesamolin after administration [43,63,64,65]. Two studies reported the bioavailability of sesamolin in in vivo models. A study by Kang investigated the effect of sesamolin on lipid peroxidation using a rat model that was fed with 1% sesamolin. Less than 25% of the ingested sesamolin was absorbed, metabolized, and excreted directly. A high level of the sesamolin in the form of its conjugated metabolites was detected in the large intestine. Only trace amounts were detected in the plasma, stomach, liver, kidneys, and small intestine. Sesamolin did not affect the body weight of the rats, but liver weight gain was found [42]. Another study by Ide reported that sesamolin altered gene expression of proteins involved in hepatic fatty acid oxidation in rats to a higher degree than sesamin but to the same degree as episesamin [66]. The sesamolin concentration in serum increased soon after oral administration, peaked at 7 to 9 h and decreased after with half-lives of 7.1 ± 0.4 h, which was longer than sesamin and episesamin (4.7 ± 0.2 and 6.1 ± 0.3, respectively). Sesamolin was highly accumulated in serum and liver compared to sesamin and episesamin. However, liver weights were also found to increase in rats given diets with sesamolin. There is no report related to the clinical study of sesamolin in humans or pharmacokinetic study in the animal. However, there is a clinical study that uses sesame seeds and oil, which contain sesamolin to investigate the effect of sesame lignans (sesamin and sesamolin) on the level of human plasma γ-tocopherol. It was reported that sesamolin and sesamin were attributed to increase plasma γ-tocopherol and inhibition of vitamin E degradation in humans without side effects [67,68].

## 7. Future Prospects

Just like other sesame lignans compounds, sesamolin was reported to have various pharmacological activities mostly tested in in vitro models. These pharmacological activities were shown towards some cell lines with low effective concentrations (<100 µM). This matter could give rise to some pros and cons. A significant effect at low concentration represents a strong activity, especially for a protective activity that does not aim to kill the cells. On the other hand, the difficulties to increase the concentration, especially in the in vitro experiments, which mostly use aqueous medium, are causing limitation in evaluating the activity or level of toxicity of sesamolin.

Sesamolin possessed low cytotoxicity against some cancer cells, e.g., SK-MEL-2 and HCT-116 [54,69]. The cytotoxicity assay of sesamolin compared with the sesamol and sesamin against SK-MEL-2 indicated that these three sesame compounds offered the potential ability to inhibit melanoma cell growth in a concentration- and time-dependent manner. However, sesamolin exhibited a low reduction in melanoma cell viability at a concentration between 50 µM to 100 µM. Only sesamol gave the 50% inhibitory concentration (IC_50_) against melanoma despite required a high treatment concentration (1893.1 ± 170.7 µM). It was mentioned in the study that sesamolin could not be dissolved well in cell culture media at a concentration higher than 200 µM that causing the limit investigation at a higher concentration [65]. These findings suggest that although sesamolin had the potency to inhibit melanoma cell growth, the limitation related to the solubility hindered the cytotoxic effect.

Another solubility problem was seen when sesamolin was tested for its in vitro extracellular antioxidant activity. Although sesamolin showed low scavenging ability towards DPPH and peroxyl free radicals, it showed higher scavenging activity towards superoxide radicals at 100 µM. The investigation at a higher concentration range could not be done due to its low aqueous solubility. Besides being caused by the fact that sesamolin’s molecular structure lacks a phenolic hydroxyl group, the solubility issue might also contribute to the difficulties to investigate the accurate antioxidant activity. Solubility issues might also be one of the reasons there have not been reports regarding the IC_50_ of sesamolin when evaluated for its cytotoxicity in vitro. Further investigation of the pharmacological activity in the in vivo model using individual sesamolin has not been widely explored, mostly in extracts that contain sesamolin.

Various strategies have been developed to overcome the physicochemical property problem that hinders the pharmacological activities of bioactive compounds. Examples of exploitation of drug delivery systems to enhance the solubility of sesamin were the formation of the micelle, solid dispersion, and nano-emulsion carrier delivery systems. The improvement in solubility, dissolution profiles, oral bioavailability, intestinal permeability of sesamin, and consequently pharmacological activities of sesamin were evident [70,71,72]. Interestingly, there is less study regarding the sesamolin’s physicochemical properties enhancement. This issue is open for further exploration and has become one of the prospective research opportunities. In addition, investigations of the pharmacological activity of this compound are still wide open, especially when the solubility problem can be resolved.

The findings of the present study suggest that sesamolin seems promising as a bioactive compound in vivo and beneficial for health. On the other hand, further clinical studies and safety studies are required. It can be clinically translated for the best use of special and differential skin treatment based on its verified antioxidant capacity and antimelanogenesis for cosmeceutical purposes, and evidence of the antitumor activity for treating skin cancer. As far as we are concerned, the literature on the investigation of sesamolin such as metabolic profiles, biological activity in vivo, and application studies is scarce. We hope that this review article could shed light on further studies to fill the gaps in this field by summarizing the current research status on sesamolin.

## 8. Conclusions

Sesamolin is one of the major lignan compounds in sesame seeds and oil and is found in a variety of sesame, white, brown, and black in various percentages. Sesamolin can be isolated and purified using chromatography techniques, then elucidated the structure using spectrophotometry techniques. Pharmacological activities of sesamolin including antioxidants, skin melanin inhibition, cell-protective effect against various stressed-induced cell death, and cancer cell killing effects via proliferative inhibition and immune stimulation. Sesamolin, therefore, can be a potential therapeutic agent against many diseases and can be explored further. Since there are few reports on the direct cytotoxicity effect of sesamolin against cancer cells, thus no publication has reported its IC_50_. Moreover, its killing mechanisms remain unclear. In addition, sesamolin pharmacological activities in the in vivo experiment and its safety have not been reported. Only allergic skin reaction is presented [73]. The underlying mechanism of sesamolin in benefiting humans is not fully evident. The problem with sesamolin could be due to its physicochemical properties, which have low water solubility. Therefore, it is difficult to increase the concentration in the in vitro experiment conditions using the cell model and will give low bioavailability in the in vivo experiment. The solubility enhancement is considered to be important for sesamolin to improve and conduct further investigation on the pharmacological activity profile. Additionally, there have been few reports studying sesamolin’s physicochemical properties enhancement; this could be explored further, becoming a prospective research opportunity in this field.

## Figures and Tables

**Table 2:** Physicochemical properties of sesamolin.

Physicochemical Properties	Properties Value
Molecular weight	370.4 g/mol
Density	1.4 ± 0.1 g/cm^3^
Boiling point	94 °C
Melting point	520.8 °C
Solubility in water	Very slightly soluble
Solubility in organic solvent	Ethanol 0.5 mg/mL
	DMSO 12 mg/mL
	DMF 30 mg/mL
Lipophilicity (log P_octanol__/aqueous_)	3
Hydrogen bond donor (HBD)	0
Hydrogen bond acceptor (HBA)	7
Topological Polar Surface Area	64.6 Å

**Table 3:** Pharmacological activities of sesamolin.

Activity	Cell/Animal Model	Effective Concentration	Mechanism of Action	References
Antioxidant	In vitro study	200–250 µg/mL	Showed weak DPPH radical scavenging ability, ferrous reducing ability power (FRAP), oxygen radical absorbance capacity (ORAC), and neutralization of the linoleate free radical in the β-carotene system.	[38,39,40]
	In vitro using rat liver microsomes	1%	Did not inhibit lipid peroxidation activity of rat liver microsomes induced by ADP-Fe^2^^+^/NADPH.	[42]
	In vitro using rat liver microsomes and rat liver mitochondria	0–100 µM	Inhibited lipid peroxidation in rat liver microsomes with cumene hydroperoxide (CumOOH)/Fe^2^^+^-ADP-NADPH lipid peroxidation system but did not show inhibition in rat liver mitochondria with Fe^2^^+−^ascorbate system.	[43]
	In vivo using rat model		Inhibited lipid peroxidation in rat liver and kidney due to metabolized form of sesamolin, which are sesamol and sesamolinol.	[42]
Antimicrobial	In vitro study	2 mg/mL	Growth inhibition against *Bacillus cereus, Staphylococcus aureus*, and *Pseudomonas aeruginosa*.	[40]
Neuroprotective	Murine microglial cells (BV-2)	50 µM	Protected cells from hypoxia damage by scavenging of ROS in hypoxia-stressed BV-2 cells followed by inhibition of MAPK signaling pathways and inhibited apoptosis.	[46]
	Murine microglial cells (BV-2)	50 µM	Protected the cells against H_2_O_2_-induced injury via reduction of ROS and inhibited p38 MAPK as well as caspase-3 preventing from apoptosis.	[47]
	Rat pheochromocytoma cells (PC12)	50 µM	Protected PC12 cells from hypoxia and peroxide (H_2_O_2_)-induced cell death by suppression of ROS generation. Inhibited MAPK and caspase-3 activation.	[48]
	Murine microglial cells (BV-2)	100 µM	Protected cells from nitric oxide (NO)-induced damage by inhibition p38 MAPK signaling pathway leading to suppression of NO production.	[49]
	Rat	20 mg/kg	Attenuated the excess generation of nitric oxide in lipopolysaccharide-stimulated rat primary microglia cells	[50]
	Worm (*Caenoharbitis elegans*)	100 μg/mL	Protective effect against Amyloid-β toxicity in both muscle and neuronal cells in *C. elegans,* mechanism need further investigation.	[51]
Antimelanogenesis	Mushroom tyrosinase	500 µM	Inhibition against tyrosinase enzyme.	[32]
	Human melanoma cells (SK-MEL-2)	50 µM	Acted as a sunscreen by blocking the UV radiation which is the melanin inducer. Reduced cellular melanin content through inhibition of the TRP-1 and TRP-2 protein that involved in melanogenesis.	[54]
	Murine melanoma cells (B16F10)	50 µM	Inhibited the expression of tyrosinase, TRP-1 and TRP-2 at mRNA and protein levels	[55]
Anticancer	Human lymphoid leukemia cells (Molt 4B)	90 µM	Showed antiproliferative effect and induced apoptosis.	[56]
	Human colorectal cancer cells (HCT116)	20 µM	Inhibited proliferation via downregulation of proteins JAK2 and STAT3 that are involved in cell proliferation. Prevented cells migration via reduction of MMP-1, MMP-2 and MMP-9 gene expression.	[57]
	Burkitt’s lymphoma cells (Raji cells)	100 μg/mL	Increased NK cell-mediated cytolysis activity by increasing the expression of NKG2D ligands on Raji cells via activation ERK signaling pathway.	[58]
	Immune cells (NK cells) and Burkitt’s lymphoma cells (Raji cells)	160 μg/mL	Increased NK cell-mediated cytolysis activity by activating the MAPK signaling pathway in NK cells.	[59]
	Dendritic cells, and NK cells interaction	5 μg/mL	Influenced dendritic cells (DCs) to promote the killing and migration activity of NK cells.	[62]
